# Interaction of genotype and environment: effect of strain and housing conditions on cognitive behavior in rodent models of schizophrenia

**DOI:** 10.3389/fnbeh.2013.00097

**Published:** 2013-07-31

**Authors:** Karly M. Turner, Thomas H. J. Burne

**Affiliations:** ^1^ Queensland Brain Institute, The University of Queensland, St. Lucia, BrisbaneQLD, Australia; ^2^ Queensland Centre for Mental Health Research, The Park Centre for Mental Health, WacolQLD, Australia

**Keywords:** background strain, cognition, enriched environment, schizophrenia, animal model

## Abstract

Schizophrenia is associated with many genetic and environmental risk factors and there is growing evidence that the interactions between genetic and environmental “hits” are critical for disease onset. Animal models of schizophrenia have traditionally used specific strain and housing conditions to test potential risk factors. As the field moves towards testing gene (G) x environment (E) interactions the impact of these choices should be considered. Given the surge of research focused on cognitive deficits, we have examined studies of cognition in rodents from the perspective of GxE interactions, in which strain or housing manipulations have been varied. Behavior is clearly altered by these factors, yet few animal models of schizophrenia have investigated cognitive deficits using different strain and housing conditions. It is important to recognise the large variation in behavior observed when using different strain and housing combinations because GxE interactions may mask or exacerbate cognitive outcomes. Further consideration will improve our understanding of GxE interactions and the underlying neurobiology of cognitive impairments in neuropsychiatric disorders.

## Introduction

Schizophrenia is a complex group of disorders in which genetic vulnerability may lead to greater sensitivity to adverse environmental conditions (Bayer et al., 1999; van Os et al., 2008, 2010; Tost and Meyer-Lindenberg, 2012). Psychiatric epidemiology has provided clues about biologically plausible combinations of genetic and environmental risk factors for the neuroscience field to examine (Caspi and Moffitt, 2006; Meyer and Feldon, 2010). For example, being raised in an urban environment has repeatedly been linked to an increase in psychotic symptoms, however this risk is amplified in individuals with a genetic predisposition to psychosis (van Os et al., 2004; Krabbendam and van Os, 2005; Spauwen et al., 2006; Weiser et al., 2007). Unravelling the neurobiological changes that lead to vulnerable or resilient phenotypes may provide important information about how gene (G) x environment (E) interactions occur and provide clues for the research community. Rodents have been used to model biologically plausible risk factors and we are beginning to appreciate the complexity of GxE interactions on outcomes relevant to schizophrenia. With the recent focus on measuring cognitive deficits in rodent models (Jentsch, 2003; Kellendonk et al., 2009; Young et al., 2009; Keeler and Robbins, 2011; Bussey et al., 2012) and the known influence of strain and housing conditions on cognitive measures (Chapillon et al., 2002; Harker and Whishaw, 2002; Wolff et al., 2002; Pena et al., 2009; Simpson and Kelly, 2011), it is important to consider whether schizophrenia-related outcomes are dependent on the strain or housing conditions used.

Currently there is a lack of animal models of schizophrenia investigating these GxE interactions on cognitive outcomes. For example, a PubMed search using the terms “strain”, “housing”, “schizophrenia”, “cognition” and “animal model” returned no results; substituting “strain” for “gene”, and “housing” for “environment” or “enrichment” only returned seven research articles although none in which housing conditions were compared. Guidelines for cognitive testing in rodents have been established to improve the progression of novel drug treatments, and the use of animal models to examine GxE interactions on established cognitive tests are needed to bridge the translational gap. The next challenge is, therefore, to develop animal models to test the hypothesis that GxE interactions affect cognitive behavior in animal models of schizophrenia. This article focuses on the consequences of strain and housing conditions on cognitive outcomes in rodent models of schizophrenia and how these factors may be useful in modeling GxE interactions.

## Modeling the cognitive deficits in schizophrenia

Cognitive deficits are a core symptom group associated with schizophrenia and are the strongest predictor of functional patient outcomes (Green et al., 2000). While cognitive remediation techniques are beneficial, current drug treatments to improve cognitive deficits are largely ineffective and the failure to translate drug findings from animal models to clinical settings has impeded progress (Pratt et al., 2012). To guide future research an initiative of the NIMH was formed, Measurement and Treatment Research to Improve Cognition in Schizophrenia (MATRICS), to make suggestions for the development of cognitive testing in animal models of schizophrenia (Green et al., 2004; Young et al., 2009). Based on the core cognitive deficits found in patients with schizophrenia seven cognitive domains were identified including working memory and attention/vigilance (Green et al., 2004). From these domains various clinical tests were selected by the follow-up group, Cognitive Neuroscience Treatment Research to Improve Cognition in Schizophrenia (CNTRICS), to be used in validating drug efficacy and to improve consistency between research groups (Carter and Barch, 2007). In order to bridge the translational gap, tests used in animal models have also been considered and selected for future use and development (Gilmour et al., 2012; Lustig et al., 2012). Domains such as verbal learning and memory cannot be translated to rodents, however processes such as attention, memory and executive control can be measured in a number of ways (Powell and Geyer, 2007) The tests selected for rodents that best reflect the cognitive constructs measured in patients include the five choice serial reaction time task (5C-SRTT) (Robbins, 2002) and sustained attention task (dSAT) for measuring attention (Lustig et al., 2012), the attentional set-shifting task (ASST) (Birrell and Brown, 2000) and reversal learning (Izquierdo and Jentsch, 2012) as measures of executive control and the radial arm maze (RAM) and delayed match to position (DMTP) task (Dudchenko, 2004), which provide the best assessment of working memory.

The CNTRICS panel reviewed the use of these tests in both rats and mice, however the selection of species and strain should be determined based on suitability for the experimental manipulation and the cognitive test being implemented (Young et al., 2009). The use of non-human primates may also be warranted where processes need to be defined differently for humans and rodents, such as in tests of working memory (Castner et al., 2004). For example, GxE interactions were examined on cognitive outcomes in an animal model of schizophrenia using catechol-o-methyl transferase (COMT) knockout mice and the 5C-SRTT (Papaleo et al., 2012). At baseline there was no effect of sex or genotype on cognitive performance, however by manipulating the inter-trial interval, measures of impulsivity were found to differ by sex and genotype. After a mild stressor, males had impaired performance in terms of accuracy and impulsivity measures and this was particularly so for males with reduced (+/-) and absent (-/-) COMT. Other measures were found to differ based on sex and genotype only after reducing motivation. This study illustrates that phenotypes based on sex or genotype may not be readily apparent, however, differences were revealed after manipulating environmental conditions. These findings are in agreement with the suggestion that genes alone do not lead to schizophrenia, but they may predispose an individual to greater vulnerability following exposure to certain environmental insults or “hits”.

## Environmental conditions and cognition

Epidemiological evidence for the role of environmental factors suggests that housing environment may be an important factor for modeling schizophrenia in rodents (Mcdonald and Murray, 2000). Housing conditions can have a significant influence on rodent behavior and have been used to induce stress or anxiety and to alter cognitive development (van Praag et al., 2000; Nithianantharajah and Hannan, 2006; Burrows et al., 2011; Simpson and Kelly, 2011). Environmental enrichment has been incorporated to enhance sensory and motor experience through the inclusion of novel objects, expanded caging and larger social groups (van Praag et al., 2000). Environmental enrichment has been linked to a number of brain-related outcomes, such as increased brain weight, increased branching and synapse formation in the cortex, increased expression of brain-derived neurotrophic factor (BDNF), glial-cell-derived neurotrophic factor (GDNF) and nerve growth factor (NGF) and increased acetylcholine levels (see review by van Praag et al., 2000). Many of these factors are likely to affect cognitive functioning, for example NGF and BDNF are both known to play a role in learning, while acetylcholine levels have been shown to correlate with attentional performance in rodents (St Peters et al., 2011). Housing conditions have been difficult to standardise across research groups, particularly when enrichment is used. Rather than viewing this noise as a nuisance, it could be seen as an opportunity to investigate how environmental conditions interact with proposed risk factors (Toth et al., 2011).

Rodents reared in more stimulating conditions often acquire tasks after fewer trials (Park et al., 1992), have reduced age-related deficits (Soffie et al., 1999; Harati et al., 2011) and recover from injury faster (Hicks et al., 2002; see Pena et al., 2009). This may indicate phenotypes are being rescued in enriched environments or that deficits only develop in a deprived environment. In some cases, such as animal models of depression, standard housing may be largely contributing to the phenotype, possibly by reducing an animal’s compensatory ability to deal with additional challenges (Brenes et al., 2009). The brain may require stimulation beyond that provided in standard housing to develop sufficient connectivity and functionality to detect higher order cognitive deficits. Whether enrichment should be considered as a therapeutic intervention or the standard conditions required for developing a “normal” brain continues to be debated (Wurbel, 2001).

## Genetic background and cognitive performance

Mutant mouse models have been used to investigate other key candidate genes linked to schizophrenia (Chen et al., 2006). Despite the availability of tasks for cognitive testing in rodents, a recent review by Arguello and Gogos (2010) did not report any mutant mouse models in which the “top 30” genes linked to schizophrenia had been tested on an attentional paradigm. Considering the need to investigate cognitive symptoms in animal models, there is an obvious gap that needs to be addressed. The genetic risk for schizophrenia is likely to be the result of hundreds or even thousands of genes of small effect (Wray and Visscher, 2010). Systematically testing each individual mutation is unlikely to replicate the disorder, nor is this approach feasible. However, specific genetic mutants may be useful for identifying the origin of cognitive endophenotypes of schizophrenia (see review by Kellendonk et al., 2009). While using single gene mutants provides information about a particular gene of interest (Papaleo et al., 2012), the polygenic nature of schizophrenia may be better modeled by comparing different strains.

Strain-dependent changes in behavior have been observed on many cognitive tasks and in response to drugs; but these changes are also dependent on the manipulation applied (Andrews et al., 1995; Schmitt and Hiemke, 1998; Mirza and Bright, 2001; Harker and Whishaw, 2002; Wahlsten et al., 2003; Zamudio et al., 2005; Higgins et al., 2007). For example, a widely used task in animal models of schizophrenia, pre-pulse inhibition (PPI) of the acoustic startle response, is a well validated test of sensorimotor gating but results are known to vary depending on the background strain (Rigdon, 1990; Glowa and Hansen, 1994; Varty and Higgins, 1994; Varty et al., 1999; Swerdlow et al., 2001; van den Buuse, 2003). Given the variability on this pre-attentive task, it is not surprising that strain differences have also been reported using more sophisticated cognitive tasks, such as the 5C-SRTT (Didriksen and Christensen, 1993; Mirza and Bright, 2001; Higgins et al., 2007; Auclair et al., 2009). These studies also demonstrate the variability between studies using the same strain, which may be due to variation in the protocol used or the source of the strain (Andrews, 1996; Karl et al., 2011).

Rat models of schizophrenia have been developed predominantly using two albino strains, however the reasons for these selections are not always obvious. Furthermore, studies of schizophrenia-related manipulations comparing rat strains are lacking. The neonatal ventral hippocampal lesion model was compared in the outbred Sprague-Dawley (SD) and two inbred strains, Lewis and Fischer 344, which differed in stress responsivity (Lipska and Weinberger, 1995). For example, SD and Lewis rats show habituation of the hypothalamic-pituitary-adrenal axis (HPA) response to a repeated restraint stress paradigm, whereas F344 rats do not habituate within or between stress-inducing sessions (Dhabhar et al., 1997). As predicted the hyper-responsive F344 strain showed greater behavioral vulnerability to the neonatal lesion, while the hypo-responsive Lewis rats showed greater resistance when both were compared to the SD strain. Thus, stress responsivity is a critical consideration both for models utilising stressful manipulations and for the interpretation of behavioral results from different strains (Faraday, 2002). Spontaneous and amphetamine-induced hyper locomotion varied across development with strain, indicating genetic predisposition has a critical role in determining the phenotype derived from this neurodevelopmental model, although cognitive outcomes were not assessed in this study (Lipska and Weinberger, 1995).

## Gene x environment interactions and cognitive endophenotypes

The focus of GxE interaction studies in animal models of schizophrenia has taken advantage of the genetic tools available in mice, comparing mutant and control animals after adverse environmental exposures such as immune activation, stress or drug administration (Kannan et al., 2013). The influence of enriched housing conditions on rodent models of schizophrenia has been addressed by only a few studies (Karl et al., 2007; McOmish et al., 2008; Ishihama et al., 2010). However, the neurological and behavioral effects of environmental enrichment have been assessed in a range of other animal models including Huntington’s disease, Alzheimer’s disease, Parkinson’s disease, Epilepsy and drug addiction (Bezard et al., 2003; see review by Nithianantharajah and Hannan, 2006; Laviola et al., 2008). For example, the influence of environmental enrichment has been shown using the transgenic mouse model of Huntington’s disease (van Dellen et al., 2000). This neurodegenerative condition has a genetic cause, yet mice housed in enriched cages show delayed onset and progression of both the motor and cognitive deficits compared to standard housed controls (Hockly et al., 2002; Nithianantharajah and Hannan, 2006; Pang et al., 2006). Using animal models of schizophrenia, it will not only be important to address the detrimental effects of the environment, but also conditions that have a protective influence (Takuma et al., 2011; Pang and Hannan, 2013).

With the aim of developing biologically-relevant animal models of schizophrenia, studies using a GxE approach are rapidly emerging (Millstein et al., 2006; Millstein and Holmes, 2007; Oliver and Davies, 2009; Desbonnet et al., 2012; Hida et al., 2013; Petrovszki et al., 2013). Prenatal stress followed by acute stress during adulthood was used in three rat strains to examine how genetic background interacted with adverse environmental conditions to alter hippocampal gene expression (Neeley et al., 2011b). Five relevant genes (*Nr3c1, Chrna7, Grin2b, Bdnf, Tnfα*) were found to be altered by either strain or stress treatments, however changes were inconsistent across strains indicating a modulatory role of genotype. A second experiment comparing these strains using a stress protocol found that changes in *Bdnf* expression and associated pathways were also strain dependent (Neeley et al., 2011a). These studies demonstrate the importance of strain selection and genetic diversity in understanding GxE interactions.

In another recent study, rats were exposed to two commonly used risk factors, post weaning social isolation and chronic ketamine treatment, and selectively bred based on behavioral deficits relevant to schizophrenia to produce a vulnerable sub-strain (Petrovszki et al., 2013). After 15 generations, four groups were compared on three behavioral tests and the results were accumulated into an overall score. Rats with a standard genetic background raised under standard conditions were used as a control group. The environment-only group consisted of genetically-naïve rats that were then isolated and treated with ketamine. Rats from the selectively bred vulnerable sub-strain that were raised under standard conditions were used as the genetic-only group. And finally rats from the vulnerable sub-strain that also underwent social isolation and chronic ketamine treatment were used to investigate the GxE interaction. The GxE group scored the highest on schizophrenia-relevant deficits and the control group scored the lowest, indicating that both genetic and environmental insults were important. The behavioral tests used assess nociception, sensorimotor gating and recognition memory, which do not address the key cognitive domains identified by CNTRICS and therefore further work would be required to understand the influence of these manipulations on cognitive deficits relevant to schizophrenia. Nevertheless, this study does present a new way of investigating previously tested risk factors.

## Future Recommendations

A recent review of mouse models of GxE interactions relevant to schizophrenia has discussed a comprehensive list of weaknesses to be addressed by future studies (Kannan et al., 2013). The authors suggested standardising strain and housing conditions to reduce variability between studies. However, genetic and environmental choices clearly alter outcomes relevant to schizophrenia and phenotypes may only be detected under specific strain or housing conditions. Furthermore, the way genetic and environmental conditions interact to protect or exacerbate phenotypes is of key importance in understanding the pathways that lead to schizophrenia.

Investigating genetic changes, such as mutant mouse models, may be easily replicated across laboratories, however environmental manipulations are more difficult to standardise. For example, wild type mice show different behavioral phenotypes when tested under similar conditions but at different laboratories (Crabbe et al., 1999). More recently, heterozygous neuregulin mutant mice showed different behavioral phenotypes when tested in different laboratories, despite being on the same genetic background (Karl et al., 2011). Although these differences may be unavoidable, it is recommended the housing conditions of rodents be clearly stated in research methods. Unfortunately many articles do not list the forms of enrichment used (such as type of bedding, shelters, wood chews and tubes) however these should be indicated even if considered to represent “standard” housing conditions. Recommending a standardised enrichment protocol would reduce variability between experiments, but would also limit the scope of enrichment studies (Wurbel, 2002). Protocol design should take into consideration the species-specific relevance of environmental changes, the timing and duration of exposure, the ethical implications and the reproducibility of the chosen design. Therefore, optimal enrichment conditions should be selected based on experimental aims.

Future studies could take a number of directions, including the use of GxG and ExE studies to identify the influence of genetic and environmental factors; as well as understanding the mechanisms that lead to increased vulnerability (Giovanoli et al., 2013). To more fully assess the effects of GxE interactions on cognitive endophenotypes, the field also needs to improve the range of the behavioral tasks available. The potential therapeutic benefit of improved animal models may be limited by the sensitivity of the behavioral measures employed. Incorporating GxE clues from epidemiology into our animal models, and improving assessment techniques will advance our understanding of schizophrenia.

## Conclusion

There is clear evidence to show that genetic and environmental conditions alter cognitive outcomes in rodents. However, the lack of studies comparing cognitive deficits in rodent models of schizophrenia using different strain and housing conditions is surprising. Schizophrenia develops from the complex interaction of GxE and we need to incorporate this complexity into animal models to understand the etiology of schizophrenia. Although it is difficult to recapitulate complex disorders, such as schizophrenia, in a rodent model, the use of endophenotypes in carefully controlled experiments may allow us to better understand some of the mechanisms behind GxE interactions. Current animal models are falling short of replicating the complex suite of risk factors implicated in schizophrenia and using different strains or housing conditions may provide an accessible stepping stone towards understanding altered brain development. Given the infancy of GxE interaction research in animal models of schizophrenia, manipulating these factors in existing and novel animal models will be informative in terms of GxE interactions. GxE interaction models will be particularly informative for understanding the role of vulnerable and resilient phenotypes in determining the influence of secondary “hits” on cognitive outcomes in schizophrenia.

## Conflict of interest statement

The authors declare that the research was conducted in the absence of any commercial or financial relationships that could be construed as a potential conflict of interest.
